# Supplementary material for: Neutralizing gut-derived lipopolysaccharide as a novel therapeutic strategy for severe leptospirosis
Source: eLife. 2024 May 31;13:RP96065. doi: 10.7554/eLife.96065 (PMC11142641; doi:10.7554/eLife.96065)
Supplement: Supplementary file 1. [file elife-96065-supp1.docx]

| Gene | Primer type | Sequence (5’-3’) | Identifier |
| --- | --- | --- | --- |
| Hamster GAPDH | Sense | GATGCTGGTGCCGAGTATGT | [1] |
|  | Antisense | GCAGAAGGTGCGGAGATGA |  |
| Hamster Claudin-2 | Sense | ACTGTCCACGGCTACCAACTACT | This study |
|  | Antisense | TCTGGCAGGGAATGTTGGAG |  |
| Hamster Claudin-3 | Sense | GCTCACCTTAGTACCCGTGTCCT | This study |
|  | Antisense | GGCAGGAGCAGCAGAGCAA |  |
| Hamster Claudin-4 | Sense | GGGGATGCTTCTCTCAGTGGT | This study |
|  | Antisense | GACACAGGCACCATGGCC |  |
| Hamster Zo-1 | Sense | GGAGAGGTGTTCCGTGTTGTG | This study |
|  | Antisense | ACTGCTCAGCTCTGTTCTTATTGG |  |
| Hamster JAMA | Sense | TGACCTGCTCCGAACAAGATG | This study |
|  | Antisense | ACTTTGGATCAACCGTGTATGAA |  |
| Hamster Mucin-2 | Sense | ATCACTTTCCAGGCCATTGAAG | This study |
|  | Antisense | CTACAGGTTCAGTTGGTCCCAGT |  |
| Hamster TNF-α | Sense | GGTGATACCAGCAGACGG | [1] |
|  | Antisense | CTTGATGGCGGACAGGA |  |
| Hamster IL-1β | Sense | TTCTGTGACTCCTGGGATGGT | [2] |
|  | Antisense | GTTGGTTTATGTTCTGTCCGTTG |  |
| Hamster IL-10 | Sense | AAGGGTTACTTGGGTTGCC | [1] |
|  | Antisense | AATGCTCCTTGATTTCTGGC |  |
| Hamster TLR9 | Sense | AGGTAAAGTGTGGCAGTCCCG | This study |
|  | Antisense | CCAAAACAATCCCAGGAAAGG |  |
| Hamster TLR7 | Sense | TTCTCCCCAACCTTGTCCAGT | This study |
|  | Antisense | ATGAAGTTAGTGCCAAGGTCAAGAA |  |
| Hamster TLR5 | Sense | GGGTCCCTGTCCCAGTATCAA | This study |
|  | Antisense | TGCCCGGAGAGTTTATTGAGAA |  |
| Hamster TLR3 | Sense | AAGCATTGCCTGGTTCGTTAG | This study |
|  | Antisense | GTATCAAACAGCATCACTGGGAA |  |
| Hamster TLR2 | Sense | TGTTTCCCGTGTTACTGGTCAT | [1] |
|  | Antisense | CACCTGCTTCCAGACTCACC |  |
| Hamster TLR4 | Sense | ACGACGAGGACTGGGTGAGA | [1] |
|  | Antisense | GCCTTCCTGGATGATGTTGG |  |
| GAM42a (Gammaproteobacteria) | probe | GCCTTCCCACATCGTTT | [3] |
| EUB338 (universal) | probe | GCTGCCTCCCGTAGGAGT | [4] |

**Reference**

1 Zhang W, Zhang N, Xie X, Guo J, Jin X, Xue F*, et al.* Toll-Like Receptor 2 Agonist Pam3CSK4 Alleviates the Pathology of Leptospirosis in Hamster. Infect Immun 2016;**84**:3350-7.

2 Zhang W, Xie X, Wu D, Jin X, Liu R, Hu X*, et al.* Doxycycline Attenuates Leptospira-Induced IL-1beta by Suppressing NLRP3 Inflammasome Priming. Front Immunol 2017;**8**:857.

3 Siyambalapitiya N, Blackall LL. Discrepancies in the widely applied GAM42a fluorescence in situ hybridisation probe for Gammaproteobacteria. FEMS Microbiol Lett 2005;**242**:367-73.

4 Snaidr J, Fuchs B, Wallner G, Wagner M, Schleifer KH, Amann R. Phylogeny and in situ identification of a morphologically conspicuous bacterium, Candidatus Magnospira bakii, present at very low frequency in activated sludge. Environ Microbiol 1999;**1**:125-35.
